# Canine circovirus genomic characterization in dogs with and without diarrheal syndrome in Medellín, Colombia

**DOI:** 10.3389/fvets.2023.1204214

**Published:** 2023-07-04

**Authors:** Diana Gomez-Betancur, Santiago Rendon-Marin, Sebastian Giraldo-Ramírez, Jairo Jaime, Julian Ruiz-Saenz

**Affiliations:** ^1^Grupo de Investigación en Ciencias Animales—GRICA, Facultad de Medicina Veterinaria y Zootecnia, Universidad Cooperativa de Colombia, Bucaramanga, Colombia; ^2^Facultad de Medicina Veterinaria y Zootecnia, Fundación Universitaria Autónoma de las Américas, Medellín, Colombia; ^3^Facultad de Medicina Veterinaria y de Zootecnia, Sede Bogotá, Centro de Investigación en Infectología e Inmunología Veterinaria (CI3V), Universidad Nacional de Colombia, Bogotá, Colombia

**Keywords:** circovirus, dogs, diarrhea, phylogenetic, coinfection, Colombia

## Abstract

Canine circovirus (CanineCV) is an emerging agent described for the first time in 2011, it infects domestic and wild canids, mainly associated with gastrointestinal signs; however, it has also been reported in samples obtained from animals without clinical signs, so its pathogenesis and epidemiology are still poorly understood. In Colombia, the CanineCV was first reported in 2020 from CPV-2 positive dogs. In the present work, CanineCV was detected in 30% of fecal samples obtained from dogs with or without diarrhea, in the city of Medellín, Colombia. No coinfection with CPV-2 was found. The highest number of positive samples was found in the subgroup of animals with diarrhea. Phylogenetic and evolutionary analyses confirmed the separation of the CanineCV genomes into five different clades with a European origin of the Colombian viruses and at least two different introductions of the CanineCV into the country. Our results highlight the importance of the CanineCV in Colombian dog populations and the need for continue surveillance of emerging pathogens in canine populations.

## Introduction

Canine circovirus (CanineCV) is an emerging agent that was first described in 2011 in the United States (1). It belongs to the family *Circoviridae* and has a covalently closed single-stranded ambisense genome of 2063 nt, with two main ORFs arranged inversely (2–4). ORF1 codes for the viral replicase gene (Rep) (nt 1-912) necessary for the initiation of the replicative cycle, and ORF2 codes for the protein capsid of the virus (Cap) (nt 1116-1928), which participates in the host's immune response (2, 5). The Cap and Rep genes of CanineCV share between 25 and 50% identity with the other known animal circoviruses, respectively (1).

CanineCV is defined as a worldwide distribution agent since its circulation has been reported on 4 out of 5 continents (6). The phylogenetic analyses of the reported strains have allowed the sequences to be classified into different clades, whose grouping depends mainly on the geographical location where each sequence was found (6). CanineCV infects wild and domestic canids, and the infection is associated with clinical pictures of acute gastroenteritis, hemorrhagic diarrhea, signs of vasculitis, lymphadenitis, thrombocytopenia, neutropenia and lymphopenia, which is related to immunosuppressive activity due to damage of the lymphoid tissue, which has been described in circovirus infection in poultry and pigs. However, the virus has also been detected in asymptomatic canine samples, so its pathogenesis and epidemiology has not yet been clearly defined (4, 7, 8).

The prevalence of CanineCV in different countries, both in samples of patients with compatible signs and asymptomatic animals, is variable, with values ranging between 1 and 30%; in most cases, it is reported in coinfections with other infectious agents where Canine parvovirus type 2 (CPV-2) stands out, reaching coinfection rates of up to 100% (9). Research suggests that its role as a pathogen may be to exacerbate clinical conditions, as has been described in Porcine Circovirus (PCV) infection in pigs, weakening the immune system and favoring the entry and spread of other viruses and bacteria. However, its participation as a coinfecting agent has not been defined (5, 10, 11).

In Colombia, the circulation of CanineCV was confirmed in 2020, and the need to increase surveillance of the virus in domestic populations was proposed with the aim to better understand its role in enteric diseases in canines, its role in asymptomatic animals or in coinfection with other pathogens (12). Given this context, the present work aims to determine the frequency and genomic characterization of CanineCV and its coinfection with Parvovirus in dogs with diarrheal syndrome in the city of Medellín, Colombia.

## Materials and methods

### Study population and sample collection

A descriptive cross-sectional study was carried out using convenience sampling. A total of 103 samples of fecal matter were collected from domestic dogs, 80 samples from canines from different centers and veterinary laboratories from October 2021 to July 2022. This was done in collaboration with the veterinary doctors, and they selected the samples according to the clinical manifestations of the patients. Fresh fecal samples were collected in sterile trays, classifying the feces according to the “Waltham” fecal scoring system between 3.5 and 5. Those values represent feces with a loss of consistency, showing blood, mucus and more than 2 days of presentation (13). In addition, 23 fecal samples from healthy dogs were collected. After collection, the samples were stored at −80°C until processing.

Animal data such as age, sex and validity of the vaccination plan were collected. For vaccination, the veterinarian verifies the vaccination card for each animal following Recommendations on vaccination for Latin American small animal practitioners (14).

Animal were classified into four groups according to the age as follows: Group I: animals of months to one year, Group II: animals of more than one year to 5 years, Group III: canines of more than 5 years to 10 years and Group IV: animals over 10 years old.

This study was approved by the Ethics Committee for Animal Experimentation of the Universidad Cooperativa de Colombia in Bucaramanga. All experiments were performed in accordance with relevant guidelines and regulations. Written informed consent was obtained from all legal guardians of the animals involved in this study.

### DNA extraction

Viral DNA extraction was performed from each of the stool samples obtained using the QIAamp Fast DNA Stool mini kit (Qiagen, Hilden, Germany) following the manufacturer's instructions. Only samples that met the appropriate amount for DNA extraction (≥ to 1 g) were processed. The quality and quantity of the extracted DNA was determined by means of spectrometry with Nanodrop™ One UV–Vis equipment (Thermo Fisher Scientific^®^). To guarantee DNA purity, for PCR amplifications, only samples with a DNA concentration >5 ng/μl and A260/A280 ratio between 1.5 and 2.1 were processed. DNA aliquots were stored at −80°C until use.

### Detection of CanineCV and CPV-2 by conventional PCR

For the diagnosis of CanineCV, a 533 bp fragment of the Rep gene was amplified by conventional PCR using a set of primers previously reported by Kotsias et al. (15). For each PCR, 25 μl of DreamTaq ™ Hot start Green PCR Master Mix (Thermo Fisher Scientific^®^), 3 ul (10 pmol) of primers “ForGenomic/Rev533” were utilized to amplify a 533 bp fragment from the Rep gene (5′-ATGGCTCAAGCTCAGGTTG-3′; 5′-CCGCACAGAACCTCCACTTC-3′). DNA used for each sample was normalized to 500 ng as measured by the Nanodrop and completed with molecular grade water to obtain a total volume of 50 μl per sample. The PCRs were performed in a Proflex™ PCR system with the following protocol: an initial denaturation step at 94°C for 3 min, 35 denaturation cycles at 94°C for 30 s, hybridization at 51.8°C for 30 s, extension at 72°C for 1 min and a final extension at 72°C for 10 min.

For the diagnosis of CPV-2, a fragment of the VP2 gene was amplified using a set of primers previously reported (16). For the reactions, the same protocol was followed as with CanineCV, except for the volume of primers. Four microliters of ParvoExt1F (5′-ATGAGTGATGGAGCAGTTCA-3′) and 4 μl of the first ParvoExt3R (5′-AGGTGCTAGTTGAGATTTTTCATATAC-3′) were used. The PCRs were carried out using the following protocol: an initial denaturation step at 94°C for 5 min, 35 denaturation cycles at 94°C for 30 s, hybridization at 50°C for 45 s, extension at 72°C for 1 min and a final extension at 72°C for 5 min.

For both viruses, DNA from a CPV-2/CanineCV positive sample (MT293520) previously obtained (12) was used as a positive control. Molecular grade water was used as a negative control. The results of the PCR amplification were visualized by 1% agarose gel electrophoresis with the SYBR safe DNA gel stain (Thermo Fisher Scientific). GeneRuler 100-bp DNA Plus Ladder (Thermo Fisher Scientific) was used as a molecular weight marker. The gels were viewed on the Gel Doc XR + ultraviolet light imaging system (Bio-Rad, Molecular imager, USA) using ImageLab software.

### Amplification of the CanineCV genome

The entire CanineCV genome was amplified by conventional PCR from positive samples using specific sequencing primers reported by Kotsias et al. (5) and Piewbang et al. (15), along with the pair of diagnostic primers (5, 15). The reactions were performed in a total volume of 50 μl, 25 μl DreamTaq ™ Hot start Green PCR Master Mix (Thermo Fisher Scientific^®^), and 3 μl of each first forward and each first reverse that make up each set. For each reaction, 19 μl of DNA was used to obtain a total volume of 50 μl per reaction.

### Sequencing and phylo-evolutive analysis

The amplicons obtained from the positive samples of genome amplification were sent to South Korea to be purified and sequenced by the company Macrogen™ Seoul-Korea. The chromatograms were analyzed and edited with Chromas v2.6 (Technelysium, Helensvale, Australia), and the consensus contigs were assembled with the SeqMan Pro platform in Lasergene Software v.15 (Lasergene INC. Madison, Wisconsin, USA). Consensus sequences were deposited in GenBank under accessions OQ377115-OQ377117. The DNA sequences were aligned using the Mafft Alignment method in Geneious Prime v. 10.1.2 (Dotmatics, New Zealand), where they were compared with whole genome nucleotide sequences of CanineCV from domestic dogs and wild canines obtained from GenBank^®^.

The phylogenetic tree was constructed under the maximum likelihood model in the MEGA™ X for Windows^®^, in which the nucleotide substitution model that best fit GTR + G + I was selected with a bootstrap of 1000.

For the phylogeography and times of divergence, the time to the most recent common ancestor (tMRCA), the geographical origin and the general spatial dynamics of the main clades of CanineCV were inferred using the Bayesian approach of MCMC in BEAUti/BEAST v1.8.430. The analysis was performed using a strict molecular clock with a constant population size. Fifteen million generations were executed to guarantee an effective population size >200 for the parameters evaluated in Tracer v1.730. The tree of maximum credibility was built with TreeAnnotator and visualized in FigTree V1.4.4

### Statistical analysis

The frequencies of CanineCV and CPV-2 in the individuals from whom the samples were collected were analyzed by descriptive statistics. The same apply for the frequency of CanineCV according to the sex of the animals, the age and validity of the vaccination plan. The association between the epidemiological characteristics and the frequency of CanineCV was determined through bivariate analyses in the SPSS™ v.2.7 program. The frequency of CanineCV was evaluated with sex and the validity of the vaccination plan using the chi-square test. According to the normality tests, non-parametric tests were used to evaluate the frequency of CanineCV with the age of the animals evaluated as significant with a value (*P* < 0.05).

## Results

In total, 103 stool samples were collected and divided into two groups: canines with diarrhea and clinically healthy canines. Because some samples did not meet the appropriate conditions for processing, such as quantity and quality of sample or sufficient DNA, a total of 90 of the stool samples were processed, 67 samples from the group of animals with diarrhea and 23 from the group of clinically healthy animals.

The epidemiological data–age, sex, and validity of the vaccination plan for both groups– are shown below in Table 1.

**Table 1:** Epidemiological data of sampled animals with diarrhea and clinically healthy.

	**Animals with diarrhea (*n =* 67)**	**Clinically healthy animals (*n =* 23)**
**Sex**		
Males	58.2% (*n =* 39)	60.8% (*n =* 14)
Female	41.8% (*n =* 28)	39.2% (*n =* 9)
**Age (by groups)**		
Group I: 0–1 year	61.2% (*n =* 41)	4.3% (*n =* 1)
Group II: 2–5 years	22.0% (*n =* 14)	43.4% (*n =* 10)
Group III: 6–10 years	11.9% (*n =* 8)	26.0% (*n =* 6)
Group IV:> 10 years	5.9% (*n =* 4)	30.7% (*n =* 7)
**Validity of the vaccination plan**		
Yes	53.7% (*n =* 36)	96% (*n =* 22)
No	46.2% (*n =* 36)	4.0% (*n =* 1)

Of the 90 samples processed, 27 positive samples for CanineCV (30%) were detected; three samples were positive for CPV-2 (3.3%), and no coinfection was detected. When evaluating the samples by clinical outcome, the group that presented the greatest number of samples positive for CanineCV was that of canines with diarrhea (*n* = 22) followed by clinically healthy animals (*n* = 4). Regarding the sex variable, male animals were most positive for CanineCV (61.5%) followed by females (38.5%).

When evaluating the age variable, the age group with the greatest number positive for CanineCV was Group I (0–1 year). When evaluating the validity of the vaccination plan, considering the reports made by the owners and/or treating veterinarians, it was found that in the CanineCV-positive canines, 61.6% had been accurately vaccinated.

To evaluate the association between the qualitative epidemiological characteristics (sex and validity of the vaccination plan and diarrhea) of the sampled canines and the frequency of CanineCV, the Pearson chi-square test was used. For the here evaluated variables, the results showed that there was no statistical association between the frequency of CanineCV with sex (*P* = 0.813) or with the validity of the vaccination plan (*P* = 0.619) nor with the presence of diarrhea (*P* = 0.1) in the evaluated animals. Besides, using the non-parametric tests, Kruskal–Wallis test, there was no association (*P* = 0.814) between the frequency of CanineCV and the age of the sampled animals.

From positive samples, we were able to amplify and sequence in good quality the full genome from only three samples (GenBank accession numbers OQ377115-OQ377117). Regarding the phylogenetic analysis, an initial search in BLAST ™ indicated that the CanineCV sequences obtained in this study showed a maximum nucleotide identity of 96.5–97% with the CanineCV-1367/2016 strain of CanineCV (GenBank: MT193166), which belongs to a strain of dogs co-infected with CPV-2 in Italy. In addition, a nucleotide identity of 96.5% was determined with one of the strains reported in Vietnam, CanineCV-VN-8 (MT740200), isolated from dogs also infected with CPV-2. In relation to the Colombian sequences identified in 2020, the strains of this research share a nucleotide identity between 96.3 and 96.5%. Finally, the three sequences obtained in the present study showed a high nucleotide identity between them (99.7–99.9% nt).

**Figure 1:**
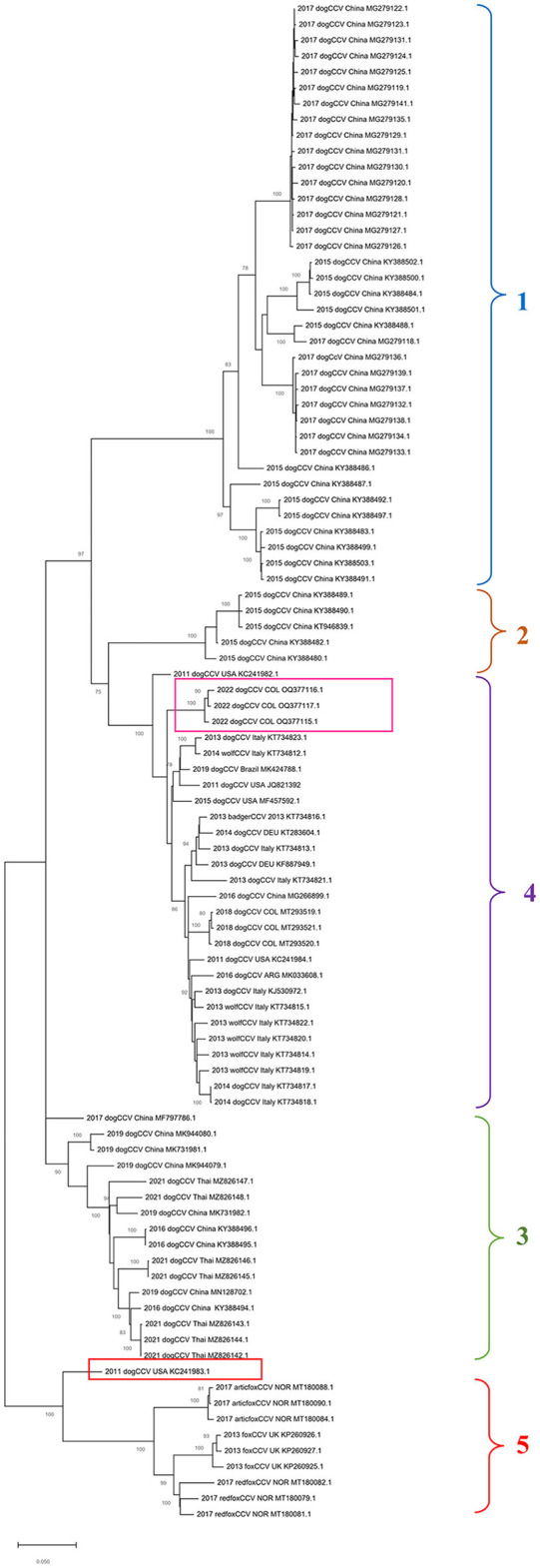
Maximum probability tree based on CanineCV whole genome sequences. The tree with the highest log probability is shown, drawn to scale, with the lengths of the branches measured in number of substitutions per site. The analysis included 79 nucleotide sequences and a total of 2,063 positions in the final dataset. The pink box indicates the Colombian sequences identified in this work, and the red box indicates the United States sequence that is not grouped in any of the clades. The phylogenetic analysis was performed using the Mega™ X for Windows^®^.

In the phylogenetic tree built based on the alignment of nucleotide sequences of the complete genome of CanineCV by the maximum likelihood method, it is possible to show the segregation of the strains in different well characterized monophyletic groups (Figure 1).

Group 1 and Group 2 exhibits sequences identified in dogs in China in 2015 and 2017; Groups 3 includes sequences from China identified between 2016 and 2019 in dogs and sequences from Thailand from 2021; Group 4, the most numerous, contains sequences of dogs identified in China in 2015, wolves and dogs from Italy, dogs in Germany and Argentina, and sequences of dogs from Colombia. The sequence identified in the United States in 2011 (GenBank: KC241983) (red box in Figure 1) was not classified in any of the mentioned groups. Finally, Group 5 contains sequences identified in fox species, which includes strains identified in Norway in Arctic foxes *Vulpes lagopus* and red foxes *Vulpes vulpes* as well as sequences identified in United Kingdom in red foxes.

**Figure 2:**
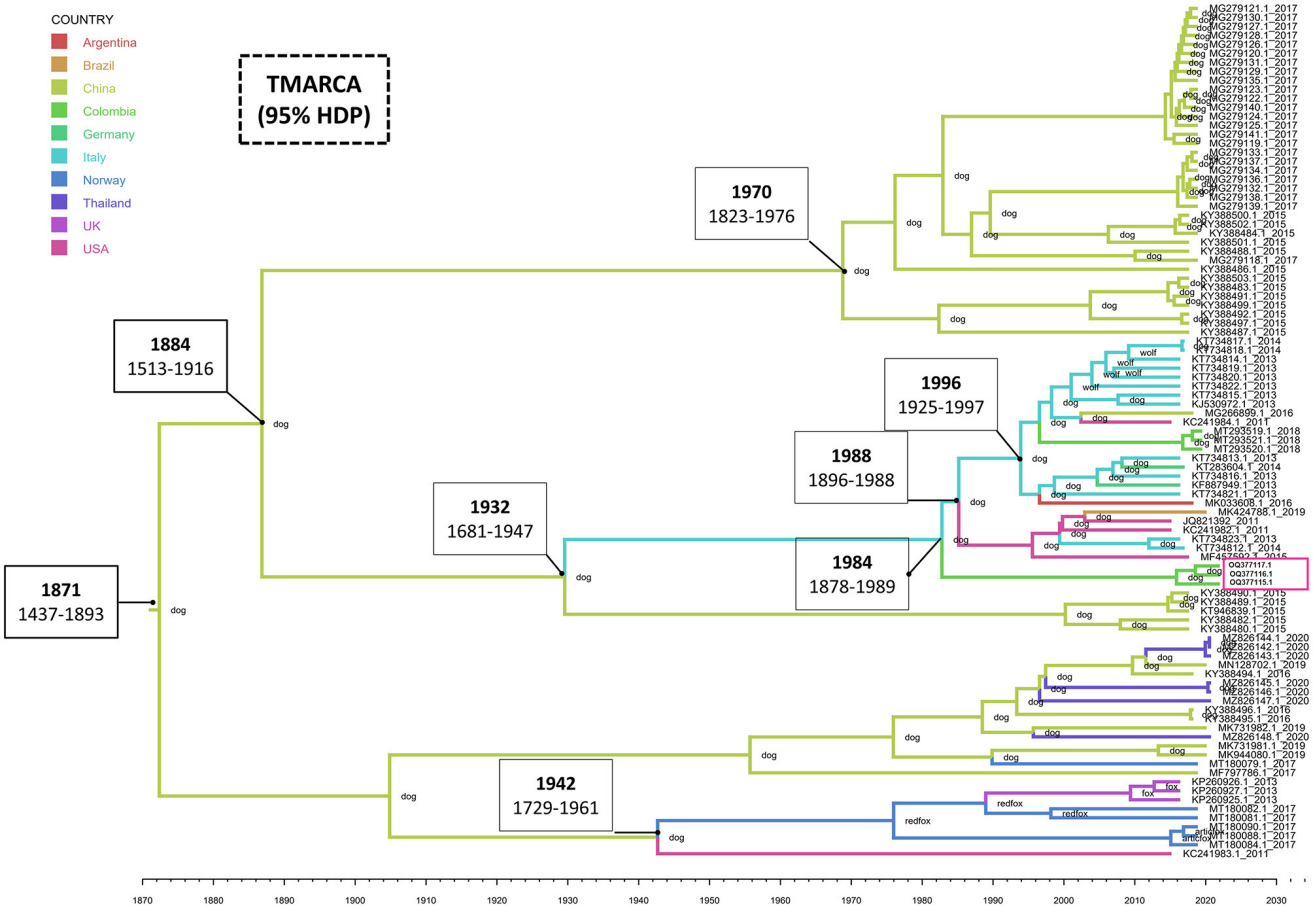
Phylogeography and divergence times of CanineCV. The tree was generated using whole genome sequences from different countries. The sequences are identified with the accession number and date of collection; in addition, each node has the species in which the sequence was identified. The timeline shows the moment of evolutionary divergence of the sequences of each country, indicating in the main division nodes the corresponding year and the HDP interval of 95%. The pink box indicates the Colombian sequences identified in this work. The MCMC tree of maximum credibility was built with TreeAnnotator and visualized with FigTree v1.4.4.

The estimated mean tMRCA for the entire CanineCV genome was 1871 (95% HDP range 1437–1893). The phylogeographic analysis shows the Chinese origin of CanineCV in domestic dogs. The Chinese sequence gave rise to the Italian sequences in 1932 (1681–1947), the United States' sequences in 1941 (1728–1960) and later moved on to the fox sequences in Norway, thus giving rise to the clade of foxes, which also includes strains identified in the United Kingdom. However, it is striking that the sequence in the United States, preceding this group, is one that to date has not been established in any clade as such (Figure 2). Although the organization of the Chinese sequences in the same clade is evident, ancestral Chinese sequences gave rise to the Italian strains, evidencing a much wider genetic distance and therefore being located in another group. Further, the strains on the American continent, including, Brazilian, Argentinian and Colombian Sequences share Italian and German origins as well.

## Discussion

This epidemiological analysis confirms the presence and relevance of the CanineCV in Colombian dog populations. The percentage of genome positive CanineCV samples in this research was 30%, which places it as the second highest result in the literature, even including clinically healthy animals. The highest percentage of positivity for CanineCV was 71% in an outbreak of enteritis occurred in a client-owned litter of dachshunds in Apulia, Southern in June 2013 (3).

Unlike the first report in Colombia, on which the detection of CanineCV was determined from samples positive for CPV-2 (12), our investigation was based on samples without a previous diagnosis of CPV-2 infection. Several studies have reported high frequencies of coinfection with CPV-2 (10, 17–20). However, CanineCV has been also reported as a single agent even when evaluating the presence of other viral and bacterial pathogens (3, 21). We highlight the role of CanineCV as a relevant agent in the study of the canine enteric disease, as has been recently discussed (6).

In the present work, it was found that the frequency of CanineCV in animals with diarrhea was higher than expected, which represents a constant in the results of CanineCV investigations, in which the highest number of positive samples corresponded to subjects who presented gastrointestinal signs mainly diarrhea, indicating that CanineCV can cause disease in domestic canines (6). However, it is important to highlight that there are other factors that can cause diarrhea in canines such as other infectious agents, inflammatory conditions, genetic and immunogenic causes, which were not considered in this work, which represents a limitation to establish a direct association between the presentation of diarrhea and CanineCV positivity.

Considering besides that another aspect that has prevented the definitive correlation of CanineCV with clinical pictures of gastrointestinal disease is that it is also found in clinically healthy animals, as evidenced in this study, where there were four positive samples in the group of canines without diarrhea (15%). Li et al. (2) in 2013 in the United States (2) established a prevalence of 11.3% in dogs with diarrhea and 6.9% in healthy dogs, and this difference was not statistically significant. In contrast, in Taiwan, it was found a highly significant correlation between the presence of diarrhea and CanineCV with a prevalence of 28.02% in dogs with diarrhea and 11.9% in healthy dogs (21). Niu et al. (19) defined a prevalence of 15.6% in dogs with diarrhea and 6.7% in healthy dogs in northeast China (19). Also in Germany, it was found a prevalence of CanineCV of 20.1% in dogs with diarrhea and 7.3% in healthy dogs (18). Worldwide data apparently show that CanineCV can be found more frequently in dogs with diarrhea, even when no significant difference between the detection of CanineCV in healthy dogs and dogs with hemorrhagic diarrhea has been reported (10).

CanineCV clinical signs in positive animals have been associated with histopathological findings such as vasculitis in the intestine and spleen, and necrosis in Peyer's patches and lymph nodes and spleen (2, 11). Such findings are similar to those reported in PCV2 infection in pigs. As a model of the family *Circoviridae* (30–33), it is well known that PCV2 predispose the pig to contract other infectious agents and increase the severity of the clinical disease by preventing adequate recovery of the animal reducing the response capacity of the immune system (22, 23). CanineCV suppresses the activation of the type I interferon promoter, promoting the replication of other viruses such as CPV-2 increasing disease severity. Therefore, coinfection between CPV-2 and CanineCV may result in more severe clinical symptoms than infection with CPV-2 alone, even in vaccinated animals (24). It is possible to propose that CanineCV could be part of the intestinal microbiota of some canines and behave as an opportunistic agent in cases of co-infection by other pathogens or factors that affect the immunity of the animal, such as stress, as has been described in pigs (22, 25) and presented as a possibility for domestic and wild canines (4, 9, 20).

Age of the infected animal can be an important factor for the pathogenesis of the disease. The diarrheic youngest animals (from birth to 1 year of age) had the highest percentage of positive samples (Table 1). This patter has also been reported by others (3, 4, 9) and has been related to the developing immune systems of young animals, which makes them more susceptible to infections (6, 20) and could also explain the highest prevalence of CPV-2 in this age group (16). However, age-related decrease in the prevalence of CanineCV disease may be due to the development of a specific immune response induced by previous infections with a progressive elimination of the infectious agent. This immune situation could explain a possible role of adult animals as asymptomatic carriers of CanineCV as recently proposed (26).

Regarding the phylogenetic analysis of the virus, the present work confirms the genotypic segregation of CanineCV into different clades, which has been reported in previous studies in Thailand, China, Taiwan, Norway and Colombia (5, 21, 27, 28). As seen in Figure 1, the sequences tend to be organized according to the geographical area where they were identified. With China origin accounting for most sequences. However, due to CanineCV still being an emerging virus, there are not enough viral sequences to fully understand the world distribution of the virus.

Phylogeny also confirms the segregation of most of the wild canid sequences to a single Clade (Clade 5 in Figure 1). In samples taken from infecting arctic foxes *(Vulpes lagopus)* in Svalbard and red foxes *(Vulpes vulpes)* in Northern Norway between 1996 and 2001(8), CanineCV DNA was detected indicating that the virus circulated in arctic foxes at least 15 years before its first discovery in sera from domestic dogs in the United States (1). Current evolutionary analysis (Figure 2) shows an ancestor of the virus in domestic dogs around 1871 (95% HDP range 1437–1893). Although it has been suggested that wild carnivores may have harbored an ancestor of CanineCV and passed it on to domestic individuals (8), our results shows the division of clades occurred in approximately 1942 (1729–1961) from an ancestor of domestic dogs, indicating that the species jump occurred from domestic to wild animals. In addition, it is possible to observe that the ranges provided by the 95% HDP interval presented in this work are quite wide, which suggests that the circulation of the CanineCV could have occurred many years ago than reported. A strong interplay between domestic and wild canids can be assumed by analyzing Codon usage adaptation of the CanineCV coding sequences (29), indicating that CanineCV is best adapted to *Vulpes vulpes*, followed by *Canis familiaris, Vulpes lagopus, and Canis lupus* undergoing cross-species transmission between them with a Bat Circovirus as ancestor (29).

Our phylogeography indicates that the species jump occurred from domestic individuals to wild species, which could have occurred in hunting dogs or human establishments (Figure 2). However, it is important to indicate that the number of sequences from dogs far exceeds that of wild canids in databases, which enter a bias inherent to the analysis in the absence of sufficient sequences of wild carnivores. Further, research in other wild canids from different locations and continents where CanineCV has been reported may help to understand the role of the interspecies transmission of CanineCV and the origin of such as has been reported for other common canine viruses such as the CPV-2 and the canine distemper virus (27, 30).

BLAST search of the Colombian sequences indicated that the CanineCV sequences obtained in this study showed a maximum nucleotide identity of 96.5–97% with the strain CanineCV-1367/2016 (MT193166), unlike the strains identified in 2020 that shared greater identity (97.53%) with the CanineCV strain Bari/411-13 (GenBank: KJ530972). While both strains come from Italy, they give the Colombian strains a different location in the phylogenetic tree, even though they share a percentage of identity with the sequences between 96.3 and 96.5%. It is possible that the paraphyletic conformation indicates different times and ancestors introduced the virus in the country, as evidenced in the result of the phylogeography where it is observed that the sequences identified in the year 2020 showed an introduction to the country close to 1997 (1994–2006), while those found in this study indicate its introduction in 1991 (1979–1999). Giraldo-Ramirez et al. (10 proposed that the transmission of CanineCV from Europe (Italy) to South America coincided with the second migratory wave to Ecuador of clade Europe I of CPV-2 that began in 2000 (27). These data allow us to propose that the entry of CanineCV occurred in coinfection with CPV-2 in this season (16, 27) which coincides with the findings of the present work that show an introduction in Colombia at the beginning of that decade in 1992 (1994–2006). Due to the low number of Colombian sequences that add a bias to the result, the fact that CanineCV in Colombia has two probably introductions at different times and probably origins, highlights the need for future research that focus on obtaining sequences of animals located in other regions of the country and in wild canids.

## Conclusions

Since the first report of CanineCV a decade ago, studies have been reported on the epidemiological and pathogenic mechanisms of the CanineCV to understand its role in enteric diseases in canines. We showed that the frequency of CanineCV is high in animals with diarrhea compared to what was expected, without requiring coinfection with CPV-2, which exposes the subdiagnosis of the virus. The phylogenetic analyses shown two different introductions of the CanineCV to Colombia and highlights the need to carry out evaluations in wild canids and in other regions of Colombia. Therefore, research in other wild canids could help to understand virus origins and interspecies transmission of this important emerging virus of domestic and wild canines.

## Data availability statement

The datasets presented in this study can be found in online repositories. The names of the repository/repositories and accession number(s) can be found at: GenBank accession numbers: OQ377115, OQ377116, and OQ377117.

## Ethics statement

The animal study was reviewed and approved by the Ethics Committee for Animal Experimentation of the Universidad Cooperativa de Colombia in Bucaramanga. Written informed consent was obtained from the owners for the participation of their animals in this study.

## Author contributions

Conceptualization, validation, resources, and data curation: JR-S and JJ. Methodology and formal analysis: DG-B and SR-M. Software and visualization: SG-R. Investigation and writing—original draft preparation: DG-B. Writing—review and editing: DG-B, SR-M, SG-R, JR-S, and JJ. Supervision, project administration, and funding acquisition: JR-S. All authors have read and agreed to the published version of the manuscript.
